# Lactoferrin as Immune-Enhancement Strategy for SARS-CoV-2 Infection in Alzheimer’s Disease Patients

**DOI:** 10.3389/fimmu.2022.878201

**Published:** 2022-04-25

**Authors:** Fernando Bartolomé, Luigi Rosa, Piera Valenti, Francisco Lopera, Jesús Hernández-Gallego, José Luis Cantero, Gorka Orive, Eva Carro

**Affiliations:** ^1^ Group of Neurodegenerative Diseases, Hospital Universitario 12 de Octubre Research Institute (imas12), Madrid, Spain; ^2^ Network Center for Biomedical Research in Neurodegenerative Diseases (CIBERNED), Madrid, Spain; ^3^ Department of Public Health and Infectious Diseases, University of Rome “La Sapienza”, Rome, Italy; ^4^ Neuroscience Group of Antioquia, Faculty of Medicine, University of Antioquia, Medellín, Colombia; ^5^ Department of Neurology, Hospital Universitario 12 de Octubre, Madrid, Spain; ^6^ Department of Medicine, Faculty of Medicine, Complutense University of Madrid, Madrid, Spain; ^7^ Laboratory of Functional Neuroscience, Pablo de Olavide University, Seville, Spain; ^8^ Laboratory of Pharmacy and Pharmaceutical Technology, Faculty of Pharmacy, University of the Basque Country, Vitoria, Spain; ^9^ Bioaraba, NanoBioCel Research Group, Vitoria-Gasteiz, Spain; ^10^ Networked Center for Biomedical Research in Bioengineering Biomaterials and Nanomedicine (CIBER-BBN), Barcelona, Spain; ^11^ Neurobiology of Alzheimer’s Disease Unit, Chronic Disease Programme, Instituto de Salud Carlos III, Madrid, Spain

**Keywords:** Alzheimer’s disease, dementia, COVID-19, SARS-CoV2, lactoferrin, saliva, brain-immunity interactions, inflammation

## Abstract

Coronavirus 2 (SARS-CoV2) (COVID-19) causes severe acute respiratory syndrome. Severe illness of COVID-19 largely occurs in older people and recent evidence indicates that demented patients have higher risk for COVID-19. Additionally, COVID-19 further enhances the vulnerability of older adults with cognitive damage. A balance between the immune and inflammatory response is necessary to control the infection. Thus, antimicrobial and anti-inflammatory drugs are hopeful therapeutic agents for the treatment of COVID-19. Accumulating evidence suggests that lactoferrin (Lf) is active against SARS-CoV-2, likely due to its potent antiviral and anti-inflammatory actions that ultimately improves immune system responses. Remarkably, salivary Lf levels are significantly reduced in different Alzheimer’s disease (AD) stages, which may reflect AD-related immunological disturbances, leading to reduced defense mechanisms against viral pathogens and an increase of the COVID-19 susceptibility. Overall, there is an urgent necessity to protect AD patients against COVID-19, decreasing the risk of viral infections. In this context, we propose bovine Lf (bLf) as a promising preventive therapeutic tool to minimize COVID-19 risk in patients with dementia or AD.

## 1 Summary

The coronavirus disease 2019 (COVID-19) pandemic is caused by severe acute respiratory syndrome coronavirus-2 (SARS-CoV-2) that attacks mainly the human respiratory system but can also access the central nervous system (CNS) (1–3). The total number of affected patients surpasses most of the health care system capacities worldwide; hence COVID19 pandemic represents an unprecedented burden for countries. COVID-19 is a multifactorious infectious disease that can lead to severe multiorgan damage and death. Among pre-existing medical comorbidities, patients with dementia have an increased risk of developing severe COVID-19 and mortality associated with it (4–8). During pre-pandemic times, patients with Alzheimer’s disease (AD) and other dementias are among the most vulnerable and dependent persons in society and this pandemic has further exacerbated their vulnerability. These observations support the need to keep safe patients with AD or dementia within the already discussed strategic plans to control the COVID-19 pandemic (8, 9).

Even if vaccines can prevent pandemic, numerous scientific investigations are considering antiviral drug therapy as an additional treatment for COVID-19 patients. Currently, a number of antivirals are in development and, some of them, such as remdesivir, showed beneficial effects reducing time to recovery (10). Another antiviral drug candidate, Paxlovid, has just received approval for FDA Emergency Use for Novel COVID-19 Oral Antiviral Treatment in USA, and very recently, EMA recommended its authorisation in the EU. The decision came following the results that treatment with Paxlovid significantly decreased hospitalisations or mortality in patients in risk to suffer of serious COVID-19.

Although large-scale vaccination is advancing around the world, effective antivirals are absolutely necessary. Antivirals that limit infection and diminish COVID-19 sings would be extremely useful to protect vulnerable patients helping to stop this pandemic. Based on this requirement, repurposing of the US Food and Drug Administration (FDA)-approved drugs is a promising strategy for identifying rapidly deployable treatments for COVID-19. In this context, lactoferrin (Lf), a glycoprotein found in secretory fluids, has been shown to inhibit SARS-CoV-2 infection, and has been proposed as a readily translatable therapeutic option for the management of COVID-19 (11–14).

Here, we offer an overview regarding the urgent need for AD patient’s protection, focusing on the inhibition of viral infection through the restoration of iron homeostasis disorders as well as improving immune system to fight viral infections, specifically SARS-CoV-2. We propose to supplement the COVID-19 standard treatment with bovine Lf (bLf), based on its therapeutic power and scientific evidence on its antiviral and anti-inflammatory activity (11–15) together with Lf deficiency at the salivary level in the AD (16, 17).

## 2 Background

### 2.1 Association Between COVID-19 and AD

Evidence supports the Theory that patients with dementia have high COVID-19 risk (8, 18). Among CNS comorbidities of COVID-19, AD stands first (19), and both diseases share risk factors, including age, obesity, cardiovascular disease, hypertension and diabetes mellitus (20). It has been suggested that pre-existing brain pathology and more specific mechanistic aspects of dementia could increase the risk of neurological complications in COVID-19 (21). Blood-brain barrier (BBB) in patients with dementia is damaged, facilitating the access of certain bacteria and viruses to the brain (22, 23) thus increasing the susceptibility to infection (24, 25). Additionally, *APOE4*, which confers increased susceptibility in developing AD, has been considered as a marker that increases the severity of COVID-19 (26) therefore, AD patients who carry the *APOE4* allele have a higher risk of developing COVID-19.

It has been already documented that patients with dementia are more susceptible to bacterial, viral, and fungal infection (24, 25, 27–30). These results included the presence of viral and bacterial DNA in post-mortem brain tissues, and/or their respective antibodies in the serum or cerebrospinal fluid (CSF). Based on all these studies, the “Infectious Hypothesis” has gained traction in recent years, which proposes that infectious agents may have a causal role in the development of AD. Moreover, and based in the close relationship between infections and inflammation, the Infectious Hypothesis presumably connects to the neuroinflammation in many ways (31). Systemic bacterial and viral infections may rise the inflammatory processes and the predisposition to develop AD (32, 33). Infectious factors are responsible for the activation of glial cells that produce several inflammatory molecules, including cytokines such as tumor necrosis factor (TNF)-α, interferon-γ (IFN-γ), interleukin (IL)-6, IL-1β, IL-18, chemokines, and reactive oxygen species (ROS) which in turn leads to exacerbation of other AD pathologies. It is important to underline that the iron homeostasis disorders, which lead to an iron overload, induce ROS formation (34).

In addition, the chronic inflammatory processes observed in AD, characterized by high levels of pro-inflammatory cytokines, can markedly influence iron homeostasis. In the brain, iron modulates different functions such as high aerobic metabolic ability of neurons, the synthesis of myelin, the synthesis and metabolism of neurotransmitters as well as the development of the neuronal dendritic tree (35). In AD patients, magnetic resonance imaging highlights an increase of iron content in the brains (36). The increase of free iron concentration in these patients, indicating a dysregulation of iron homeostasis, compromises brain functions due to the increase of oxidative stress associated with higher ROS and nitric oxide synthase (NOS) production (37–39). In the absence of inflammation, iron homeostasis guarantees a correct distribution of this metal between tissues/secretions and circulation. Every day 15 mg of iron are ingested from the diet but only 1-2 mg of iron are daily absorbed (34).

Therefore, in inflammatory processes must be taken into account that high levels of hepcidin and low levels of ferroportin (40) cause an iron overload in cells and secretions together with iron deficiency into the blood (41). These peptides are able to modulate iron homeostasis. It is well known that patients suffering from neurologic disorders such as AD or other kind of dementia, showed systemic metabolic disorders such as anemia or anemia of inflammation (42). Conversely, low levels of hepcidin and high levels of ferroportin, restore iron export thus decreasing intracellular iron load and increasing iron in the blood (43, 44).

Of note, viral infections are directly promoted by intracellular iron overload because their replication is dependent from host cell iron enzymes, some of which are involved in transcription, viral mRNA translation, and viral assembly (45). As AD patients are characterized by high concentration of intracellular iron (36), they have an increased replication rate of COVID-19 with the consequent neurological and systemic complications.

SARS-CoV-2 enters the body mainly as droplets during inhalation, and infiltrates the nasal and buccal cavities to gain access to the mucosa and the respiratory tract. But SARS-CoV-2 may also entry into the brain across the CNS vascular barriers (46). Pathogens can access to the CNS by several ways and possibly speed up the progression of AD. The first is through a compromised BBB. In a healthy situation, BBB provides a selective barrier to the passage of cells and molecules into the brain; however, in a pathological situation, a compromised BBB can allow direct entry into the brain through the passage of blood to the CSF (23, 24). Moreover, pathogens, including bacteria and viruses can bypass the BBB by entering *via* the olfactory system, because the nasal cavity connects the peripheral environment to brain regions such as the olfactory bulb, the entorhinal cortex and the hippocampus (47). SARS-CoV-2 infection is widespread in epithelial cells, particularly in the lungs, starting its invasion and entry into the respiratory tract. However, as with other viral infections, SARS-CoV-2 may enter the brain by its neuroinvasive properties, directly by infection of olfactory sensory neurons in the epithelium and then transported into the CNS through the olfactory nerve, or crossing the BBB (46, 48).

It is known that SARS-CoV-2 uses angiotensin-converting enzyme 2 (ACE2) as a receptor to enter the host cells and a recent article reports that ACE2 levels are upregulated in AD brains (49). Although some studies proposed that there was no clear evidence for human neuronal or astrocyte expression of ACE2 (50), more recent findings report robust ACE2 expression in human neurons, which is a target for SARS-CoV-2 infection (51). Moreover, Aβ_42_ binds to ACE2 and the spike protein S1 subunit of SARS-CoV-2, enhancing SARS-CoV-2 infection/inflammation (52). Additionally, viral infection reciprocally affects Aβ_42_ clearance, amplifying the progression and severity of AD. These results may indicate a higher risk of viral entry and loads in the brain in these patients, contributing to understand the relationships between COVID-19 and the brain, particularly in AD.

Most groups in this field have focused their research on vulnerability of people with dementia to SARS-CoV-2 infection because their impaired memory impedes them to comply with the suggested public health recommendations. Two attention-grabbing papers discussed this pandemic situation and its impact on demented patients. In one of them, Mok and colleagues discussed the worrying impact of COVID-19 upon patients with AD and other dementias, and proposed strategies for care and management of these patients and their caregivers (9). More recently, a retrospective study of adult and elderly patients in the United States up to 2020 showed that patients with dementia were at higher risk for COVID-19 than those without dementia, and interestingly they found black people with dementia had higher risk of COVID-19 than white people (8). Results of this study highlight the need to keep safe patients with AD or dementia, placing special emphasis on the black people, within the pandemic control.

In both studies, authors pointed out the particular vulnerability because the multiplicity of medical conditions and social/environmental factors. However, in Wang’s study, authors speculate that preexisting brain injury may allow more virus entry inducing the pathology of COVID-19 within the brain (8), but, they also draw attention to a poor immune response/immune dysregulation as others studies reported (53, 54).

Thus, an effective and robust immune response may face more effectively the outcomes of the SARS-CoV-2 infection (55). It is proposed that dysregulated immune function, including impaired antimicrobial function, is associated with increased susceptibility to infections (56).

**Figure 1:**
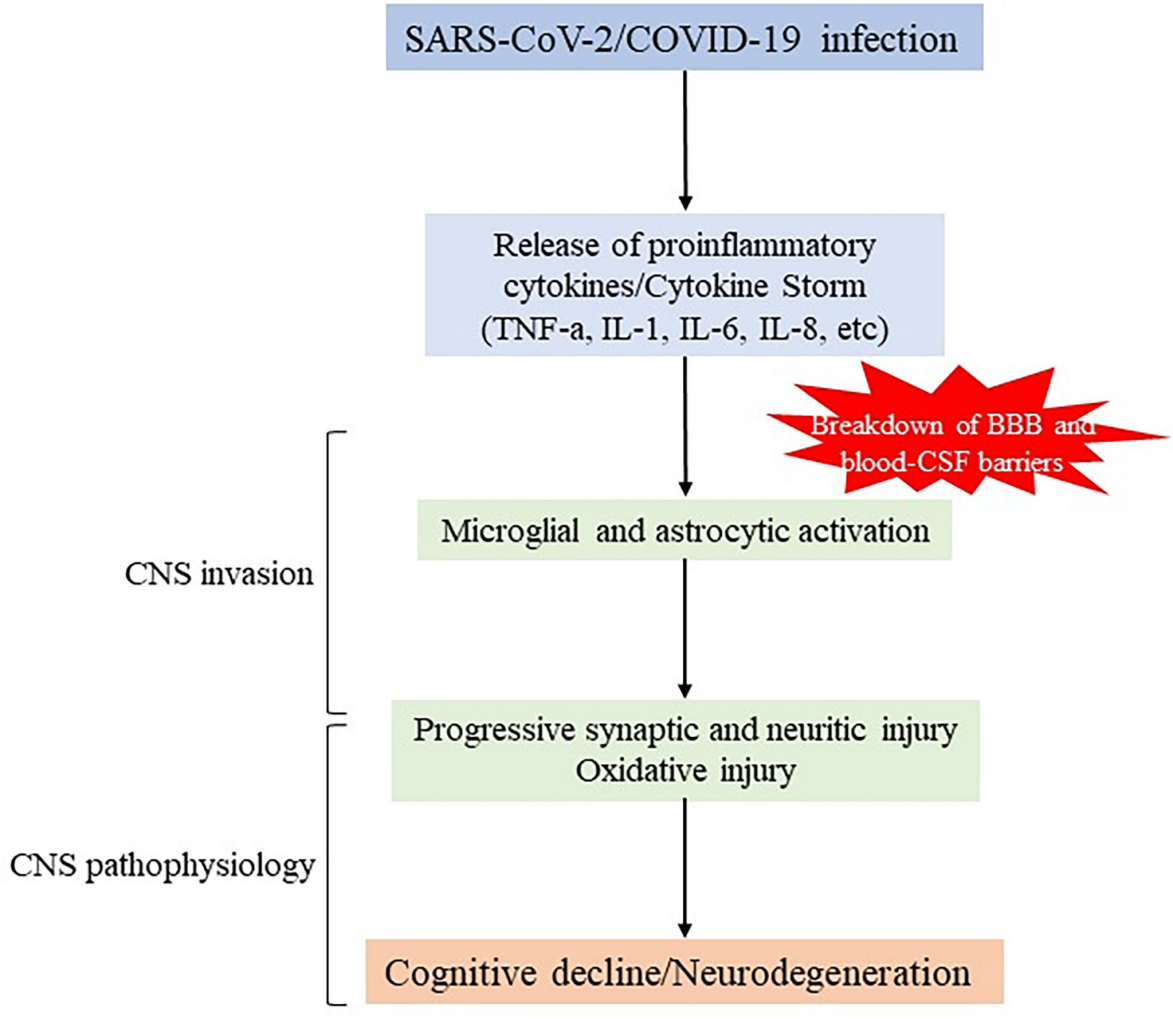
Schematic representation of potential mechanisms of SARS-CoV-2 infection in neurodegeneration. SARS-CoV-2 infection causes severe upregulation of proinflammatory cytokines and chemokines (so called “cytokine storm”) leading to increased permeability of BBB and blood-CSF barrier, and initiating CNS invasion This event also involves overactivation of glial cells that can promote detrimental effects, indirectly and/or directly, by inducing synapse loss, oxidative injury and further contributing to neuronal degeneration.

As has been widely seen, patients suffering for severe COVID-19 develop high levels of proinflammatory cytokines and acute respiratory dysfunction. These inflammatory processes have been suggested to cause cognitive decline (32, 57, 58). Pathogenically, this situation may result from direct negative effects of the immune reaction, exacerbating of pre-existing cognitive deficits, or *de novo* induction of neurodegenerative disease (**Figure 1**).

Understanding the relationship between immune dysregulation, infections and dementia has taken on new urgency in the COVID-19 pandemic.

### 2.2 Impact of the Immune System in AD

The bidirectional pathways between the CNS and peripheral immunity are called the neuroimmune axis, and even if are far from being completely understood (59–62). Neurological inflammation disorders are thought to be caused by dysregulated afferent nerve neuroimmune pathways (62, 63). Regarding the possible roles that may play in the pathology of AD the dysbiosis or infections outside the CNS, there are limited data. However, given the role of innate immunity in AD has become clear in recent years, as well as the connection between the neuroimmune axis and neuroinflammation, we believe that more attention should be paid to the contributions of chronic peripheral infection on cerebral AD pathology.

As we recently discussed, the evolution of AD pathology is associated with immunity dysfuntion (56). Alterations in the immune responses may occur at early stages of AD and possibly are involved in the AD progression, as reported in previous experimental and clinical studies (64). Throughout aging, there is a loss of anatomical and physiological integrity, which causes a greater vulnerability to some diseases and death. Aging is induced by genetic, epigenetic and environmental factors and affects almost all organs, having a profound impact on the immune system (65, 66). Moreover, several studies postulated that AD pathology is under the control of the immune system in an age-dependent manner (67–69). Now, AD is consider a systemic disease with a strong central and peripheral neuroinflammatory component. Immune cells may travel to and from the brain due to the increased permeability of the BBB in AD, participating in the pathogenesis and progression of this neurodegenerative disorder (69). Growing evidence suggests that peripheral infections may trigger the build-up of amyloid plaques in the brain by modulating glial cells, eliciting an immune reaction and stimulating secretase activity that increase the production of amyloid peptides (70, 71). Over the last decade, the presence of a sustained immune response in the brain has been proposed as a key element in AD pathology. Neuroinflammation, including the activation of glial cells and other immune cells, has been demonstrated to aggravate AD–related pathology (72). Acute inflammatory responses are common to healthy individuals, however chronic inflammation impairs the natural balance of pro- and anti-inflammatory signaling in the brain, presumably leading to the development and progression of neurodegenerative diseases such as AD (72).

The innate immune system induces an essential control over salivary secretion in the oral cavity. The nasal cavity is the major portal of entry for pathogens as well as the oral cavity which homeostasis is maintained by saliva. The most relevant salivary agents responsible for the defense against microbial pathogens are antimicrobial peptides and proteins (AMPs), which are the primary innate immune effectors and constitute the first line of defense against pathogen invaders (73, 74).

In humans, AMPs are produced by many cells, including phagocytes, epithelial and endothelial cells (75). Particularly, AMPs are highly expressed in the brain and other immunoprivileged organs where the activities of adaptive immunity are constrained, and low AMP levels can result in seriously compromised immunity. AMP expression can be induced during inflammation or after microbial infections. It has proposed that dysregulation of AMP activities may be involved in the pathology of chronic inflammatory diseases and neurodegenerative disorders (76). The normal production of AMPs may be reduced as a result of factors such as a debilitated immune system, damaged defense cells (by either intracellular infections or apoptotic processes), or structural vitamin D deficiency (77). Down-regulation of AMPs is associated with chronic inflammatory diseases, including Crohn’s disease (78).

As previously discussed, the innate immune system utilizes AMPs as the primary effector proteins to attack invading microorganisms, such as bacteria, viruses or fungi (79). By binding host biomolecules linked to immunity, AMPs are also potent immunomodulators that play an important role in combating infection. AMPs modulate both innate and adaptive immune systems which normally work as a continuum (80). In fact, AMPs are sometimes known as alarmins due to their role in stimulating adaptive immune pathways, including the complement system.

#### 2.2.1 Salivary AMPs in AD

Recent discoveries on inflammation-mediated neurodegeneration and the role of Aβ in immunity have led to the emerging of the “Antimicrobial Protection Hypothesis” in AD (81). AMPs have been proposed as a potential alternative for the detection and diagnostic follow-up of such cerebral infections that affect the Aβ accumulation in the brain (82). Furthermore, antimicrobial therapies could also be effective in attacking AD pathology (83).

The host-response consists of a cascade of events by the innate and acquired immunity. An early component of the host response is the secretion of AMPs by salivary glands, oral epithelial cells and neutrophils. Saliva provides valuable information on oral and systemic health. Most of the salivary compounds are locally produced in the salivary glands, but some others can come from blood, such as secretory IgA, transported by active transport, ions, catecholamines, and steroids, transported by ultrafiltration mechanism, or plasma albumin which enter saliva by transudation into the oral cavity (84). Saliva significantly contributes to the protective barrier of oral epithelium through its content of AMPs, which may have an important role in innate host defense. Saliva contains a large panel of antimicrobial proteins including Lf, lactoperoxidase, lysozyme and antimicrobial peptides that both directly or indirectly inhibit the uncontrolled outgrowth of pathogens. Although the concentrations of some of these molecules are quite low, their effects are additive and/or synergistic, constituting an efficient molecular defense system of the oral cavity (85).

Salivary proteins, including AMPs, are released from salivary glands under the autonomic nervous system control through the release and activation of acetylcholine (ACh) from parasympathetic nerves (86–89). The primary parasympathetic salivary centers connect with the lateral hypothalamus. Development or immune disorders may originate salivary gland dysfunction. We suggested together with other authors that AD-related immune system disturbances might be a result of neurological alterations determined by hypothalamic lesions (56, 60, 90). Reinforcing this hypothesis, we have recently reported reduced ACh release measured in submandibular glands from APP/PS1 double-transgenic mice model of AD (91). Additionally, we found lower salivary Lf levels in this mouse compared with non-transgenic mice, suggesting a specific dysfunction in the AD salivary glands associated with an altered ACh signaling pathway. These findings are consistent with those previously reported in AD patients showing lower salivary Lf levels in prodromal and clinical AD (16, 17).

#### 2.2.2 Role of Salivary Lf in AD

Lf, an 80 kDa iron-chelating cationic glycoprotein belonging to the transferrin family, exerts several functions, such as antibacterial, antifungal, antiviral, antiparasitic, anti-inflammatory and immunomodulatory activities (34, 92–95). Human Lf (hLf) is expressed in a variety of tissues and fluids including breast milk, colostrum, saliva, tears, mucous, as well as it is present in the secondary granules of neutrophils (96–98). HLf is one of the major proteins in all exocrine secretions, including saliva, which is associated with host defense against oral pathogens. The concentration of salivary hLf in healthy subjects ranges between 3.9 and 14.5 µg/ml (99) with mean values of 8.96 and 7.11 µg/ml in unstimulated and stimulated saliva, respectively as has been reported by authors (100). Salivary hLf concentration is also influenced by gender (34), age (101, 102), and the physiological or pathological status of the subject (99, 103–105). Concerning the pathological status of the subjects, a cross-sectional study showed that salivary hLf levels are decreased in patients suffering from mild cognitive impairment (MCI) and AD compared with age-matched healthy subjects (16), indicating a putative link between AD, the immune system, and brain infections. In addition, salivary hLf has been proposed to be useful to detect MCI or prodromal AD and to discriminate AD from other type of dementias, as salivary hLf levels are associated with the amyloid-PET imaging profile (17). Moreover, it has been just reported that salivary hLf is negatively associated with regional Aβ load and worse memory (106). Based on all these results, we support the role of salivary hLf as a biomarker of cerebral vulnerability in physiological aging. In addition, we suggested that salivary levels of hLf could be reduced as a consequence of the immunological disorders associated with AD. Moreover, changes in systemic immunity during AD progression could be a downstream effect of early AD pathology (56).

Salivary hLf is involved in the regulating of the oral microbiota and the inflammatory state of the oral mucosa, contributing to the preservation of symbiosis in the host-microbiome relationship (107). Therefore, when salivary hLf levels decreased, as seen in AD patients, it would be expected that there would be an advance of oral dysbiosis. Even in aged subjects with oral dryness, salivary levels of hLf were reduced and this may aid the access of oral pathogens to the brain (101). In addition, Olsen and Singhrao proposed that salivary hLf deficit may act as an activator of oral microbial dysbiosis, supporting the concept that low levels of hLf might indicate oral dysbiosis (108).

Other studies have also supported that oral pathogens could degrade hLf (109, 110). This could facilitate the proliferation of some of these pathogens, augmenting oral infections, and probably promoting AD by systemic dissemination of these pathogens and the inflammatory signaling in the brain. In an elderly person with deteriorated BBB, oral microorganisms and inflammatory mediators can reach the brain through the blood stream. Therefore, as Olsen and Singhrao proposed, it is highly plausible that low salivary hLf levels could promote the propagation of oral-related microorganisms and inflammatory molecules to the brain by reducing innate immunity (108).

Low salivary levels of hLf in AD patients may affect its brain concentration since salivary hLf may be transported into the brain *via* the sublingual route (111). As matter of fact, Lf can easily cross the BBB because Lf receptors (LfR) are present on the membrane of BBB endothelial cells (112), thus exerting its multiple functions. Interestingly, under pathological conditions, such as AD, an increase of LfR expression on microvessels and neurons has been reported (113). As consequence, a rapid Lf uptake by LfR and high availability in the brain have been observed (112).

#### 2.2.3 Iron Chelation Agents or Lf in AD Treatment

In the last years, as iron burden exerts an important role in the AD pathology, iron-chelating compounds got a lot of attention. However, the entry of drugs into the brain is restricted by the BBB. Therefore, an iron-chelating agent, ideal for treating AD, must easily pass through the BBB. For this purpose, nanotechnological approaches have been studied (114) together with methods of intranasal administration (115). Among iron-chelating compounds, deferoxamine (DFO) showed beneficial effects in experimental studies, as shown after intramuscular, oral or intranasal administration in AD patients (116–118). However, DFO shows a poor bioavailability and induces some side effects such as neurotoxicity in prolonged treatments as well as gastrointestinal malabsorption (39, 117–119).

Differently from iron-chelating compounds, Lf administration seems to be active against anemia or anemia of inflammation as well as against AD (120). Lf has been found to revert iron homeostasis illnesses induced by inflammatory processes by down-regulating IL-6 and hepcidin and up-regulating ferroportin expression, reestablishing iron export from cells into blood (41, 121–124). Moreover, Lf in macrophages is also able to up-regulate TfR1, and down-regulate cytosolic ferritin (125, 126).

Therefore, Lf through its potent anti-inflammatory activity and its efficacy in modulating iron protein as ferroportin, TfR1, ferritin and hepcidin is emerging as a natural substance that can be applied in the prevention and cure of anemia without side effects (124). Interestingly, Guo and colleagues (2017) reported that intranasal recombinant hLf (rhLf) treatment reduces Aβ aggregation and cognitive impairment in an AD mouse model. Furthermore, this rhLf treatment protects the brain from oxidative stress, showing decrease significant reduction of ROS, TNF-α and IL-6 levels in the brain (127).

In another clinical trial, the bLf treatment leads to a decrease of IL-6 and an increase IL-10 in serum (128). Lf has been proposed to be beneficial in AD patients, since this multifunctional protein may alleviate the AD pathological cascade by reducing Aβ-aggregation, tauopathy, spread inflammation and oxidative stress, and neuronal damage (120, 128, 129). Based on all these studies, bLf supplements are currently considered as a plausible therapy for AD.

### 2.3 BLf in COVID-19 Treatment

Since the immune status plays a crucial role in disease severity, immunotherapies are used in severely ill COVID-19 patients (130). Based on the anti-inflammatory, anti-viral and immune-regulating properties of bLf (95), which are in accordance with the treatment supplies for SARS-CoV-2 infection, bLf might be useful in the prevention and/or management of COVID-19.

Indeed, bLf could exert multiple functions, including a primary defense factor against mucosal infections, and a modulator of viral infectious processes. Its antiviral activity is mediated by the Lf binding to heparan sulphate glycosaminoglycans (HSPGs) of host cells, viral particles or both (93).

Recently, many *in vitro* studies shown that bLf is active against SARS-CoV-2 (11, 12, 131, 132). In these studies, bLf shows antiviral activity against SARS-CoV-2 with a multimodal mechanism: (i) through its binding with HPSGs of host cells, which blocks the transport of viral particles to the high-affinity specific entry as ACE-2 (131); (ii) through its binding to spike glycoproteins of SARS-CoV-2, thus hindering the viral adhesion to host cells surface (12); and (iii) through the upregulation of interferon I system thus activating the host antiviral response (11, 132). All these findings propose the beneficial bLf effects in host defense against SARS-CoV-2.

As reported in the prestigious *Lancet* journal, mortality from COVID-19 is not simply due to viral infection but is a result of a cytokine storm syndrome associated with hyperinflammation leading to acute respiratory distress and subsequent mortality (133). By the way, many studies shown that bLf was able to modulate this cytokine profile in COVID-19 cases by reducing reduce IL-6 and TNFα levels (126, 134–138). Moreover, bLf may diminish inflammatory factor release by promoting different actions (139). It was reported that after oral administration of bLf, the killing activity of NK cells was higher against virus-infected cells, enhancing the production of IL-18 (140). Also, bLf may rise IL-12 levels in macrophagocytes, promoting the migration of macrophages to inflammatory sites (141).

As Zimecki and colleagues summarize in their recent review, several studies strongly suggest the utility of bLf to silence the “cytokine storm”, supporting its potential for the handling of SARS-CoV-2 infection (142).

Based on preclinical studies, Rosa and colleagues developed a recent study to assess the efficacy of oral bLf on ambulatory COVID-19 patients (13). Results of this study revealed that the time required achieving SARS-CoV-2 RNA negativization in bLf-treated patients was lower compared to that reported in bLf untreated patients (15 versus 24 days). This means that the bLf treatment may improve outcomes in patients suffering from COVID-19, including those with comorbid diseases, and advanced age. Furthermore, they detected a very interesting association between symptom reduction and age: bLf was able to reduce the time to symptom resolution with advancing age (13). This fact could be associated with the hormonal control of hLf synthesis (143), and that decreases with age. The latter is particularly relevant for AD patients showing lower salivary hLf levels (16, 17).

In another clinical study in Tor Vergata University Hospital (Rome, Italy), oral and intranasal liposomal bLf was administered in asymptomatic and mild-to-moderate COVID-19 patients compared to standard of care (SOC)-treated and untreated COVID-19 patients (14). In agreement with previously reported data (13), significantly less mean time to SARS-CoV-2 RNA negative conversion was detected in a liposomal bLf-treated group compared to SOC-treated and untreated patients (14 versus 27 days), with fast clinical symptoms recovery (14). Moreover, in liposomal bLf-treated patients, a significant reduction in serum IL-6, ferritin and D-dimers levels was shown (14).

Overall, even if the randomized clinical trials on a large number of COVID-19 patients are required, these clinical experiences indicate that early treatment of bLf on COVID-19 patients could be the winning strategy to avoid the disease progression and severity, especially in the patients suffering from comorbid diseases and advanced age.

## 3 Challenge in AD Patient Protection Against COVID-19

Although we have gained a greater understanding of the impact of COVID‐19 on dementia, and particularly on AD, further research is urgently needed to protect these vulnerable patients against COVID-19, controlling the risk of viral infections. Implementing prevention of infection and surveillance measures is vitally important. Studies aimed at reduce the adverse response of COVID-19 infection in AD patients to with a dual approach including both, early detection programs, where the primary health system will be implicated, and therapeutic interventions will be helpful and necessary.

Although at the time of writing there are a number of antivirals in development as potential treatments for COVID-19, and a few of them are already approved, we propose that an additional approach focused on preventing the risk of infection would be very useful. Our strategy would be focused on those vulnerable populations to COVID-19 infection, specifically AD patients, with impaired innate‐immune defenses. They would be easily detected by using a very simple and non-invasive test (i.e., measuring salivary Lf levels).

**Figure 2:**
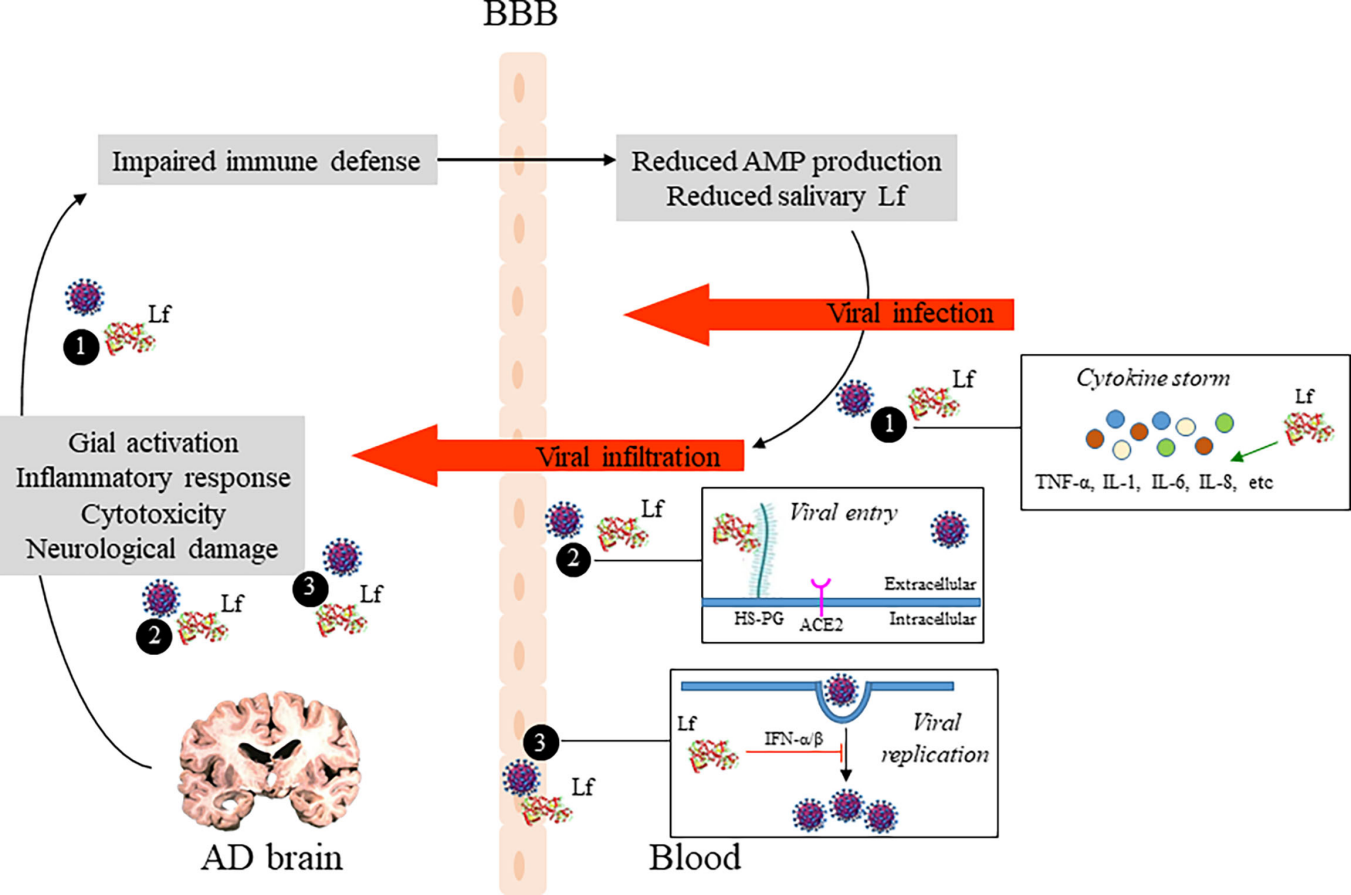
Potential role of Lf in the relationship between AD brain pathology and COVID-19. Pathogenic events leading to neuronal damage may impair the host defense system which in turn reduce AMP production, including Lf, and influence the extent of SARS-CoV-2 infection in the brain. Additionally, potential antiviral mechanisms of Lf are shown: (1) by modulating SARS-CoV-2 induced inflamation, reducing pro-inflammatory cytokine levels, such as IL-6 and TNFα; (2) by occupying binding sites of SARS-CoV-2, as heparan sulfate proteoglycans (HSPGs) on the host cell surface, reducing viral surfing and subsequent viral entry; and (3) by inhibition of viral replication *via* induction of intracellular cell signals. AD, Alzheimer’s disease; Lf, lactoferrin; AMP, antimicrobial peptide; BBB, blood-brain barrier.

Here, we propose a double strategy aimed at reducing the vulnerability of AD patients against SARS-CoV-2infection by enhancing immune system: the first approach would be to measure salivary hLf levels and the second would be to restore antimicrobial defense including bLf supplementation, which would reduce SARS-CoV-2 actions based on the multifunctional properties of bLf (i.e., antiviral and anti-inflammatory activities). As discussed by Bermejo and colleagues, AD pathology is closely related to the immune system that may be reflected as an impaired innate‐immune response (i.e., reduced AMPs production, including salivary Lf) (56). Salivary Lf production is influenced by age. We and others revealed reduced Lf activity and levels in healthy elderly subjects, being significant in the fourth decade (144–146). Interestingly, this reduction is exacerbated not only in AD-diagnosed patients (16, 17), but also in memory impaired subjects associated with brain Aβ burden (106). Although more functional studies to analyze in the CNS the consequences of altered salivary Lf levels in AD would be necessary, it is close clear that reduced salivary Lf levels may be used to identify demented and cognitive impaired people due to AD susceptible of infection by SARS-CoV-2 (**Figure 2**). Our strategy could be especially useful in less developed countries where COVID-19 but also AD incidence are growing and have devastating consequences for their population.

The need to implement standards and protocols aimed at preventing of SARS-CoV-2 infection is crucial to avoid or at less reduce greater risk of COVID-19 morbidity and mortality. In this regard, we propose to introduce control strategies in the primary care health system focused on monitoring the immune status of AD patients and other vulnerable people with or without dementia (i.e., subjects with subjective memory loss). Because of the pressure on the sanitary system caused by the COVID-19 pandemic, analytical methods to evaluate the immune status should be fast, relatively cheap, and should not require specialized personnel. In the fluid biomarker field, salivary biomarkers provide a rapid and efficient disease diagnosis. With the evidence that Lf represents an important defensive element as a modulator of the immune response, we propose that salivary Lf could be a useful tool for the screening of these vulnerable older people. Additionally, further studies are needed concerning molecular and functional links between salivary immune biomarkers and AD neurodegeneration addressing questions like, when do these immune alterations appear?; do they appear before or after the first clinical symptoms of dementia?; how long do they remain?

Moreover, cumulative evidence support the anti-viral activity of bLf which interacts with cell HSPGs and SARS-CoV-2 spike glycoproteins, thus appearing a hopeful alternative for the treatment of COVID−19 (**Figure 2**). As widely supported by Zimecki and colleagues, bLf may be of clinical benefits in the decreasing of the SARS-CoV-2- induced cytokine storm (142). We suggest that salivary Lf decrease may be a result of impaired innate‐immune defenses, and consequently the elderly would be more susceptible to infections. The clinical experiences reported by Rosa et al. (13) and Campione et al. (14) indicate that early treatment of bLf on COVID-19 patients could be the winning strategy to avoid the disease progression and severity, especially in the patients suffering from comorbidities and advanced age.

AD patients who are more prone to COVID-19-related deaths due to immune dysregulation and iron homeostasis disorders are likely to benefit from Lf supplements. Nevertheless, we recommend further clinical studies to validate Lf in AD patients against COVID-19 infection to prove its efficacy in overcoming a hyper-inflammation status and cognitive impairments, thus reducing mortality. In addition, it is possible that Lf supplements can also be supplied as a preventive treatment for those vulnerable older people as a pharmacological strategy to reinforce their immune response. However, more knowledge is needed to determine if this strategy is truly protective and which are the events and molecular pathways involved.

## 4 Conclusions

Here we examine evidences for the relationship between SARS-CoV-2 infection and AD supporting that people with this neurodegenerative disease have an increased risk for viral infection, probably dependent from an iron overload, an inflammatory process and a deficient immune response. As an antiviral agent, Lf works directly or indirectly on the viral particles and is being used for several health purposes. At this time, more than 170 clinical trials include bLf. Since bLf represents an easily available and safe natural glycoprotein, it may become a new preventive approach to help the vulnerable population, including AD patients suffering from COVID-19. However, questions still remain as to whether bLf therapeutic intervention could avoid SARS-CoV-2 infection. Future studies should consider evaluating these aspects through preclinical and subsequent clinical trials. We propose testing the levels of salivary Lf in the population at risk (AD patients and/or healthy older subjects with subjective memory loss) and planning interventions to raise its levels. It is important to underline that different bLf formulations are commercialized: orosoluble tablets, intranasal spray and oral capsules could be the winning strategy to increase salivary Lf levels and to protect AD patients from SARS-CoV-2 infection. The use of orosoluble tablets and the intranasal spray is active against SARS-CoV-2 through bLf binding with cell HSPGs and viral spike glycoproteins, with hinders viral infection. These strategies should incorporate randomized, placebo-controlled, parallel-group Phase-1 clinical trials to determine in these participants the preliminary efficacy and impact of bLf on COVID-19. Well-designed clinical studies are needed to further validate the use of bLf as effective treatment in the SARS-CoV-2 infection.

Although COVID-19 vaccination is working successfully, in the current scenario we strongly believe nutraceutical supplements, including Lf, appear to be promising alternative solutions for the prevention and treatment of COVID-19.

## Author Contributions

EC: conception and design of the study, drafting the manuscript and figures. FB: drafting the manuscript. LR and PV: drafting and critical review of the manuscript. FL: drafting and critical review of the manuscript. JC: drafting and critical review of the manuscript. JH-G: drafting the manuscript. GO: drafting and critical review of the manuscript. All authors have read and agreed to the published version of the manuscript.

## Funding

This study was supported by grants from Instituto de Salud Carlos III (FIS21/00679 to EC and JH-G and PI21/00183 to FB), FEDER, Comunidad de Madrid (S2017/BMD-3700; NEUROMETAB-CM to EC), and CIBERNED (CB07/502 to EC); Spanish Ministry of Economy and Competitiveness (PID2020-119978RB-I00 to JC), CIBERNED, the Research Program for a Long-Life Society (0551_PSL_6_E to JC), the Junta de Andalucía (PY20_00858 to J.L.C.), the Andalucía-FEDER Program (UPO-1380913 to JC).

## Conflict of Interest

EC and GO are co‐founders of GEROA Diagnostics. FL was supported by COLCIENCIAS-Colombia (111565741185), and Genentech/Roche/API COLOMBIA GN28352.

The remaining authors declare that the research was conducted in the absence of any commercial or financial relationships that could be construed as a potential conflict of interest.

The reviewer EM declared a shared affiliation with the authors LR and PV to the handling editor at the time of review.

## Publisher’s Note

All claims expressed in this article are solely those of the authors and do not necessarily represent those of their affiliated organizations, or those of the publisher, the editors and the reviewers. Any product that may be evaluated in this article, or claim that may be made by its manufacturer, is not guaranteed or endorsed by the publisher.
